# Styles of thought in healthcare governance: A situational analysis of English PrEP discourse 2016–2020

**DOI:** 10.1057/s41292-025-00351-8

**Published:** 2025-04-13

**Authors:** Adam Christianson

**Affiliations:** Department of Sociology, Goldsmiths, University of London, London, UK

**Keywords:** HIV prevention, Molecularisation, Style of thought, Governmentality, Grounded theory

## Abstract

Attending to competing styles of thought in healthcare controversies may be helpful to critical health scholarship. This article reexamines the debate over the introduction of a new HIV prevention technology in England as a tension between epidemiological and molecular style of thoughts. I argue English HIV services were organised according to an epidemiological style of thought. The introduction of biomedical pre-exposure prophylaxis (PrEP) to the health system brought this rationality into question in ways the English health system was ill-prepared to manage. A situational analysis of English PrEP discourse in the lead up and following NHS-England’s ‘U-turn’ on PrEP illustrates a split along epidemiologically and biomedically informed styles of thought. These networks have their dedicated administrators, experts, activists and ways of thinking about their target population and preferred organisation of HIV services. Though they often collaborate, these two groups have distinct moral and political agendas that relate to their style of thinking. This analysis further nuances existing critical interpretations of the PrEP controversy in England. Beyond England, this debate suggests a potential departure from the conventional biopolitical subject and rationality of advanced liberalism.

## Introduction

The social implications of biomedical interventions are often eclipsed by their promise. Biomedical interventions are often ambivalent technologies, the beneficial effects of which are shaped by contrasting cultural ideals of health, illness, and responsibility (Persson 2004; Gaspar et al. 2022; Auerbach and Hoppe, 2015; Epstein 2007). Likewise, identity is increasingly shaped by biomedical visions of health and vitality (Rose 2007; Clarke et al., 2010; Rabinow 1996) and is an increasingly important site of politics (Rose 2007; Rose and Novas 2005; Rose and Rabinow, 2015). As biomedical knowledge filters into our relationships with self and others, it comes into contact with more established biopolitical techniques. To address this hybridisation of scientific and political identity, incorporating the *styles of thought* (cf. Fleck 1979; Rose 2007; Hacking, 2002) linking experts, authorities, activists and their subjects can help analyse such debates.

This article considers how biomedically informed thinking conflicts with established knowledge and techniques in HIV prevention. The success of HIV prevention initiatives has mainly been attributed to the mobilisation of gay and other men who have sex with men (MSM) as biological citizens (Rose and Novas 2005). In the absence of a vaccine and with treatments in development, MSM created community-based, epidemiologically informed HIV prevention methods (Crimp 2003; Epstein 1996; Patton 1990; Boellstorff 2011). Though these methods would be refined over the decades, behavioural HIV prevention remained largely unchallenged until evidence from randomised clinical trials in the late 2000s began demonstrating that HIV-negative persons could successfully prevent HIV by taking similar combinations of antiretrovirals to persons on HIV treatment (Baeten et al. 2012; Grant et al. 2010)*.*

This novel HIV prevention method, PrEP (pre-exposure prophylaxis), allows people to prevent HIV by taking HIV medications in advance of contact with HIV. Should they encounter HIV through, for example, condomless sex with a person who is HIV-positive and not virally suppressed, the medications taken in advance and after contact deactivate the virus, allowing the immune system to eliminate infected cells without further spread.

PrEP has been heralded as something of a medical marvel. If taken consistently, PrEP is 99% effective (Grant et al. 2010). It is also more consistent, less cumbersome, and arguably more pleasurable than alternative sexual HIV prevention methods, with few, generally tolerable side effects. However, PrEP remains polarising in HIV prevention circles and amongst its intended users. Despite the evidence, scepticism, and moralising attitudes from governments, some HIV organisations and within the target community resounded throughout the 2010s. Though experts and activists in sexual health have largely denounced these positions, and others have illustrated the moralising attitudes and non-scientific thinking that seemingly fuel such perspectives (Calabrese and Underhill 2015; Haire 2015; Golub 2018), it seems there is a powerful reluctance to acknowledge or accept an evidence-based intervention. Gaspar et al. (2022) argue the triumphant optimism for PrEP overshadows many longstanding biopolitical ambivalences introduced by PrEP to HIV care.

This article reexamines the controversy that delayed PrEP implementation in England from 2016 to 2020. The English saga typically starts with a rather abrupt change of heart on PrEP prioritisation. Amid an otherwise routine review process following a highly successful open-label trial (McCormack et al. 2015a, b), the English National Health Service (NHS) issued a press release claiming it was not the responsible commissioner for PrEP (NHS 2016b). The announcement the NHS would not implement PrEP reportedly came as a shock to most stakeholders, who perceived the NHS’s change in position as a cynical attempt at ‘passing the buck’ for an expensive, complicated, and politically contentious intervention to local authorities (Paparini 2021; Khan et al. 2023; Kmietowicz 2016; Hawkes 2016a, 2016b; Iacobucci 2016). In response, United4PrEP, a coalition of mostly gay men, grassroots activists, third-sector organisations and healthcare providers took on the NHS to advocate for PrEP provision (Portman 2017; Nutland 2017). Though United4PrEP ultimately succeeded in making PrEP freely available through the NHS in 2020, this ‘U-turn’ instigated a national controversy, two judicial reviews and an ‘implementation trial’ that delayed PrEP commissioning on the NHS by nearly half a decade.

Others have cast this protracted controversy as a clear example of anti-scientific beliefs (Nagington and Sandset 2020; Dodds 2021; Paparini 2021), reflective more so of liberal and conservative politics than the evidence. However, this view oversimplifies the complex set of relations that made this colossal failure of HIV service provision possible and, as I will discuss, neglects the effects of the discourses deployed to resolve it.

Critical scholars have been more successful in illustrating the ambivalences of the PrEP debate reflective in homonormative (Mowlabocus 2019; Maine 2019) and biomedicalising (Young et al. 2019; Martinez-Lacabe 2019) aspects of UK HIV discourse; however, these interpretations often come to conflicting conclusions Jones et al. (2020) have argued that such frames obscure the deeper ambivalences within identity and biomedicine that shaped the PrEP controversy. In response, others have examined the controversy as an instance of biosexual citizenship (Jones et al. 2020; Orne and Gall 2019). Whilst biosexual citizenship is defined as the mutual shaping of sexual and biological identity and politics (Epstein 2018), these approaches tend to focus on the former more than the latter. Yet the biopolitical tensions are plentiful. PrEP causes multiple paradoxes in healthcare provision (Krakower et al. 2014; Calabrese et al. 2018; Grace et al. 2018; Holt 2015; Race, 2018; 2016). However, the biopolitical tensions underlying these paradoxes have only been discussed piecemeal.

Style of thought, introduced by Ludwig Fleck (1979), can help illustrate the tensions between evidence and governance arising in PrEP discourse. Fleck demonstrated scientific progress is not driven by the best available evidence but is often stunted and involves selective interpretation, sometimes ignoring contrary evidence due to the historical and discursive constraints on their interpretive abilities. Such constraints on thinking, likewise, affected clinicians, policymakers, and individuals in England, who needed to adopt a new perspective for PrEP even to be intelligible as prevention. The tensions between biomedical and population-based approaches to HIV prevention are underexamined in critical analyses of HIV, that often conflate epidemiology and biomedicine (Adam 2011; Mykhalovskiy and Namaste 2019), obscuring a longstanding competition between these two approaches for dominance in medical governance (Hanemaayer 2019; Marks 2008; Daly 2005; Norton 2017; Bluhm and Borgerson 2011).

## Thinking with styles of thought

An important feature of styles of thought that distinguishes them from discourses is that they designate a field of intelligibility. A style of thought refers to the possibilities for thought and action informed by a particular scientific rationality and in which “explanations are only possible and intelligible within that way of thinking” (Rose 2007, p. 12). They do not only facilitate a given means of explanation, but frame what is even there to explain. Consequently, a given style of thought tends to give prominence to a particular set of statements, explanations, and solutions as preeminent over others. However, a style is not limited to a discourse, a specific discipline or methodology. Thought styles, as I am employing them, refer to regularities in discourse in which a set of ontological, epistemic, and normative commitments become the predominant as the means of explaining, organising around, and acting on a given problem.

Styles of thought bring together what Fleck (1979) termed *thought collectives*, groups of people who think about a given problem along similar lines. This collective is broader than a discipline, as such groups involve a whole array of scientific experts, technicians, political and professional associations as well as lay persons outside the conventional boundaries of any one discipline (for a longer discussion, see Rose 2007 pp. 27–30). What characterises this collective are the regularities in their commitments to certain fundamental truths about possibilities for action, intervention, and acceptable constraints imposed by the style. These regularities reflect the commitments that align disparate actors and groups towards a similar goal. This perspective is useful, given the increased hybridisation of political and scientific approaches in activism (Guta et al. 2014; Rabeharisoa et al. 2014) and policy (Taylor 2005; Howlett 2009) across disciplinary and political lines.

Considering the styles of thinking employed in the PrEP debates in England reveal how PrEP caused the *functional overdetermination* of English HIV services (Foucault 1980). These arise when new populations or groups force existing systems of relations into resonance or contradiction, necessitating their reconfiguration (pp. 194–195).

Integrating the style of thought that underpins the alliances formed within England around HIV treatment and prevention illustrates how introducing PrEP a conflict, not only between progressive and reactionary political groups— as has already been well documented, but divided integrationist and radical reformers within the progressive camp. I explore how two networks restructured the English health system to accommodate PrEP and its users, shifting the predominant style of thinking about HIV in England.

Attending to the driving rationality that brings together and mobilises these collectives is a promising method for understanding technological and scientific controversies. As I will discuss, this approach allows analysts to reexamine the UK PrEP debate as a conflict between over two distinct rationalities. The English PrEP saga reflects two fundamentally distinct ways of thinking about what it even means to ‘prevent’ HIV. On one hand, there is a more conventional approach to HIV prevention, in which prevention is understood and acted upon between bodies, though behavioural modification and barrier methods, such as condoms and in which HIV infection is understood as a binary, instantiated by a transmission event. This approach is complemented with a biomedical approach in which HIV transmission is understood as a process, with multiple points of molecular intervention within the individual body.

The idea that HIV is prevented by preventing transmission between individual bodies belongs to an *epidemiological* style of thought. Though there are a variety of schools of epidemiology, a key feature of epidemiology, generally, is the link forged between health outcomes and populations. An epidemiological style of thought reflects a broader tendency to frame health and vitality in terms of the knowledge and techniques forged at the outset of the nineteenth century that take population as its object (Reubi, 2018; Wahlberg and Rose, 2015; Foucault 1980, 2008) and in which people are taken as self-entrepreneurs who invests in their bodies with the interest of future returns in biological capital (cf. Kenny, 2015; Foucault 2008). As opposed to conventional clinical or biomedical approaches to health, this approach uses highly sophisticated sociological and statistical techniques to explain health behaviours, beliefs, and outcomes, tends to make inferences using large amounts of data about a given population and relies on individuals to make good choices (cf. Hunt 2003; Dean 1998).

Epidemiological thinking has radically transformed the object and aims of clinical examination without compelling clinicians to abandon the methods of clinical observation. Clinical epidemiology, later EBM, emerged at a time when biomedical techniques and patient activism posed a challenge to medical authority (Marks 2008; Epstein 2007). This approach promised to preserve clinical authority by providing clinicians with an objective means to evaluate evidence and apply that evidence to their patients (Daly 2005; Hanemaayer 2019). Notably, this transformation did not require clinicians to *become* epidemiologists but to couple their ‘somatic’ observations (cf. Foucault 2003; Rose 2007) with the predictive power of epidemiology. Epidemiology has been equally crucial to HIV prevention, where infection is not immediately observable. Using statistical and sociological techniques combined with clinical observation, a clinician, other healthcare specialist can confidently determine whether their patient is infected with HIV or their likelihood of becoming infected in the future through risk calculus based on exposure type, location, and social categories without relying exclusively on biomedical testing.

Conventional HIV prevention practices have been argued to be squarely within this style of thought (Adam 2006, 2011). Since the early HIV epidemic, we have explained HIV infection as caused by certain behaviours enacted by specific kinds of self-contained individuals with certain biological and social traits (Flowers 2001). This behavioural approach to HIV surveillance and prevention has come to shape hybrid biological and sexual identities, such as that of ‘MSM’ (Boellstorff 2011). Finally, the prevalent strategies of prevention available to individuals (condoms and behaviour change) were focused on the ‘molar’ (Rose 2007) interactions between bodies and organs.

It is worth noting that this style of thought also determines the boundaries of what is intelligible as a risk. Neither sociological, statistical, nor clinical observation methods are well-equipped to address individual risk-reduction techniques. HIV prevention has been largely resistant to harm reduction techniques, such as negotiated safety or strategic positioning because it relies on relational risk-reduction techniques and embodied knowledge that is not readily explained in terms of population (Halperin 2016; Race, 2018). It also obscures some of the more nuanced aspects of HIV prevention. Because the focus is on kinds of people, transmission and infection are framed as a binary: you are either infected or uninfected, obscuring the more complex biomedically enhanced identities, such as ‘HIV-positive, untransmissible’ as a result of an undetectable viral load (Race 2001).

Molecular biology shifts perspective on HIV care by focusing on interactions at the molecular level rather than the sociological or behavioural level. Unlike epidemiology, which abstracts from populations, biomedicine delves into the mind and body's inner workings, linking HIV to brain activity and chemical flows, making them accessible for intervention according to what Rose (2007) has termed a *molecular style of thought*. From this perspective, HIV is a radically different object. Microbiologists tend to look at HIV transmissions between cells taking place within rather than between bodies. At this scale, HIV is not transmitted by sexual contact but through a transcription and replication process, replete with unique sites for intervention. Whilst it would be incorrect to say this perspective can see HIV infections better, it is undoubtedly the case that microbiological explanations are increasingly seen as more definitive and biomedical expertise is more authoritative on HIV infection.

From this biomedical perspective, there are two kinds of HIV infection: a local mucosal infection, which the immune system can handle on its own and the immune system destroying systemic HIV infection, which we are more familiar with. Seeing HIV infection from this perspective looks more like a process composed an initial stage where HIV enters a cell, its eventual reprogramming and reproduction in the cell, the progression to a local infection (usually in a mucosal membrane) and its eventual spread to the entire immune system (see, Haase 2005, 2010). PrEP capitalises on a weakness in HIV’s reproduction stage that allows it to effectively deactivate the virus once it integrates itself into an infected cell (García-Lerma et al. 2010)—the meaning of prevention shifts from avoiding contact with HIV to stopping an existing infection from progressing. Thus, PrEP is not prevention in the conventional sense—it does not keep HIV out of the body but prevents its replication once present. PrEP is, nonetheless, highly effective at preventing onward infections, outperforming methods relying on behaviour change and condom use (cf. Grant et al. 2010; Smith et al. 2015; van den Boom et al. 2014). This liminal quality makes PrEP a useful case study in examining the role of styles of thought play in healthcare governance. People must adopt unconventional ways of understanding and approaching prevention to realise its biomedical potential.

Governmentality studies is closely associated with analyses of neoliberalism, in which people are encouraged to act responsibly by exercising prudent individual choices (Rose and Novas 2005; Garland 1996). Others have illustrated the homonormative (Mowlabocus 2019) and biomedicalising (Jones et al. 2020) tendencies in the reactionary and progressive dimensions of PrEP discourse along these lines. PrEP disrupts traditional HIV prevention strategies, which rely on responsibilisation and the deliberate assessment of one’s HIV status and risk (see Adam 2005). However, others have argued that people’s decision-making abilities are naturally diminished during sex, undermining these strategies (Herron 2016, 2020; Rendina 2015). Due to its particularly high efficacy, PrEP can act in place of the responsibilised subject during such ‘hot’ moments, allowing them to act responsibly, when their rationality is diminished (Golub 2018; Herron 2016, 2020). This approach takes people as inherently fallible, with bounded rationalities (Pykett, Jones and Whitehead, 2017) whose inherent irrationalities require managed freedom (cf. Pellandini-Simányi & Conte 2021). In this respect, the debate over PrEP speaks to a broader biopolitical struggle over the subject.

## Methodology

Clarke et al. (2018) have proposed *situational analysis* as a corrective to some of the methodological limitations of grounded theory. This approach examines the material relationships, discourses and groups that together create a social phenomenon holistically. This is done using multiple concurrent *relational*, *social worlds* and *positional* maps. These maps chart the relations, alliances, and activities of discursive communities involved in a debate over time. The following discussion uses a modified social world and positional mapping strategy using the 5W1H Method to account for the styles of thought constituting the English controversy on PrEP.

Situational analyses use positional maps to trace discourse elaboration over time (Clarke et al. 2018; Friese 2010)*.* These maps trace how different discursive actors make claims in their terms about their empirical object. Conventionally, positional maps locate the significant positions taken or not taken in a debate on a coordinate grid along two axes from the weakest to the most extreme positions. However, this approach does not offer a way to analyse variation *within* the positions taken. The claim, in this case, that persons at risk of contracting HIV need PrEP and that it ought to be provided to those persons as part of the health service is shared by those actively producing discourse but had multiple contrasting and overlapping positions that did not neatly fit on this grid.

Building on Bowker and Star’s (2000) interpretation of co-production, Friese (2010) proposes analysing schemas. She argues that classifying in scientific discourse is a process that involves the imbrication and contestation of social hierarchies. When classifications are contested, such as in the case of scientific chimaeras, the interpretive heuristic undergirding the positions taken on that object should also be considered. Building on Friese (2010), I focused on the style of thought that informed people’s positions when making claims during this period. I used a variation of the 5W1H Method, a common method of structuring narrative in Western countries, to systematically assess and categorise the positions taken in discourse (see also Rose et al. 2006, p. 3). I determined the object (what is PrEP) and subject (who is it for), its ideal place in the health system (where), and its function (how it works) for each position taken in discourse. I found two general tendencies: I considered those who tended to think about PrEP as an intervention for people who belong to risk groups, where PrEP is part of a diverse toolkit that reduces risks and in which clinical judgement is privileged. PrEP works by blocking HIV from entering the body— it is more epidemiological in style. By contrast, those who tended to see people who take PrEP as part of a seamless continuum with others on treatment, where PrEP is part of a more homogeneous biomedical toolkit that prevents infections and in which individual choice is privileged. PrEP works by preventing onward transmission I, categorised as molecular.

I extended Friese’s (2010) approach to examine how styles of thought informed the alliances people formed with experts in healthcare professions and administrators. Grounded theory emerges from the idea that social life is a mosaic of groups with shared affiliations, traditions and ways of living and thinking. Situational analysis conceives these groups as discursive and material communities, mapped using ‘social worlds and arenas maps’. These maps chart the alliances and relationships between the most and least active group and individual discourse producers (Clarke et al. 2018). It also allows us to see groups involved in a debate “at a distance” (Rose and Miller, 1992), such as the Department of Health and Social Care, which abstained from the PrEP controversy but whose involvement could be felt through its alliances as well as the kinds of knowledge they drew on to make their claims authoritative, but who were not directly involved in the debate.

Alongside state, juridical and healthcare actors, two organisations emerged as the major contributors to English PrEP discourse: the Terrence Higgins Trust and PrEPster. These two organisations are the most active and arguably most influential members of a far longer list of organisations and individuals who formed the United4PrEP coalition of healthcare and third-sector actors who advocated for PrEP (see Portman 2017). The National Aids Trust (NAT) and Iwantprep.com (IWPN) were also highly active. However, the NAT became less active after seeing the NHS in 2016, and IWPN merged with the THT in 2018. Hence, NHS, THT, and PrEPster represent the most active and longstanding debate members.

In addition to producing the majority of the discourse on PrEP at this time, these PrEPster and the THT represent *evidence-based activists*, activist communities who combine traditional forms of political representation with expertise in the scientific and policy fields in which they advocate (Rabeharisoa et al. 2014). These activists combine experiential/community and scientific knowledge to represent their communities, more clearly illustrating their involvement in a given style of thought. As I will demonstrate, s PrEPster and the THT act as nodes in their networks, helping translate (cf. Rose 1999) between experiential and scientific discourses.

## The U-turn: tensions at the interface of two styles of thought

A situational analysis of the positions taken before the NHS U-turn suggests PrEP needed to be more fitted to the English health system. Apart from its efficacy, people engaging with PrEP in England worked hard to make it fit, and a slow realisation of its incompatibility with the style of governing HIV imposed on the NHS. Epidemiological rationalities shape the laws and regulations governing healthcare provision in England and inform how the NHS determine whether to prescribe a commissioned service. Healthcare is available at low or no cost to all residents of England through a commissioning process overseen by the NHS. The commissioning process allows primary healthcare providers to bill for treatments provided free to the public. Whilst the English public views the NHS very positively, successive governments have progressively stripped back its services since the 1980s (Greer 2016; Nicholas 2012). Consequently, the NHS faces the evermore challenging task of rationing increasingly diverse and costly interventions based on efficiency, cost-effectiveness, and need.

Whilst PrEP is highly effective at preventing HIV and is an invaluable tool for HIV prevention, PrEP fails commissioning thresholds. Early analyses of PrEP found that the intervention would result in a net loss for the NHS for each quality adjusted life-year (QALY) gained (Ong et al. 2015). This is the first key instance of epidemiological thinking. The QALY is a modification of the modes of epidemiolocal calculation developed over the nineteenth century to make them amenable to economic calculation (Wahlberg & Rose, 2015). It compares the average quality and quantity of years gained with the intervention to those without. The emergence of these metrics coincides with the rise of clinical epidemiology as the paradigmatic mode of medical decision-making (Hanemaayer 2019; Daly 2005). However, as Novas (2015) notes, due to the small population, specialised treatments are typically not found to be cost-efficient using QALYs. To accommodate HIV treatment, the NHS has specialised procurement arrangements that allow them to be purchased at lower cost. However, these arrangements did not extend to PrEP as it did not constitute HIV treatment. The expense, coupled with the larger population of persons at risk for HIV expanding treatment would create, has been speculated to be the main reason for the U-turn (Paparini 2021; Hurley 2019; Dodds 2021).

However, PrEP can be made cost-effective if one changes how QALYs are calculated. Rather than calculate the individual benefit of a given treatment aggregated across the population, dynamic models that found PrEP potentially cost-effective define health benefits to include averted *onward* transmissions (Cambiano et al. 2018). This means that the benefit measured included those not taking the drug. Hence, the key population is not the persons taking conscious steps to prevent HIV but a more elusive non-user who indirectly benefits from PrEP. However, conceiving this group is challenging as it brings notions of vulnerability and agency into tension (Grace et al. 2018; Holt 2015). As Race (2018) notes, PrEP extends HIV prevention to populations concurrently to people who explicitly reject HIV prevention aims or judge themselves as ‘not much of a risk’. These individuals have consciously appraised the value of PrEP and determined they do not need it. This onward benefit, though paradoxical from the perspective of conventional neoliberal conceptions of calculative individuality, is in line with notions of a notion of the subject whose calculations are limited by biology, particularly neurochemistry (Rose 2007; Pykett, Jones and Whitehead, 2017; (Pellandini-Simányi and Conte 2021) a position that will become increasingly compelling leading up to 2020.

These tensions also informed the *moral value* of PrEP. The subjects who benefit from PrEP are conventionally cast as careless, biased or deviant (Halperin 2016). Hence, there was concern at the time that overconfidence in PrEP’s efficacy would interfere with the ecosystem of collective practices and social services that sustained HIV prevention (Rosengarten and Micheal 2009; Herron 2016; Calabrese and Underhill 2015). This concern is reflected in recommendations the NICE published shortly after the U-turn:

PrEP is only one of several prevention tools for HIV, and early diagnosis through testing, antiretroviral therapy for HIV-positive people to reduce the risk of onward transmission, correct and consistent condom use, and addressing the wider determinants of poor sexual health among this population are also important.

There is little doubt that Truvada is effective in reducing HIV acquisition in high-risk people who are HIV-negative. However, issues relating to uptake, adherence, sexual behaviour, drug resistance, safety, prioritisation for prophylaxis and cost-effectiveness are also important to consider, especially at a population level. (NICE 2016)

The NICE is pitting PrEP’s biomedical efficacy against the sociological, behavioural, and economic factors that contribute to HIV prevention. Their argument here is that PrEP’s known efficacy does not make it superior to other HIV prevention tools and certainly should not be a replacement for the social supports that sustained HIV prevention, especially when the real-world effects of PrEP outside of a trial are unknown. According to one report, such concerns triggered the NHS U-turn (NHS 2016a, p. 4).

Whether these concerns were valid has been addressed elsewhere (Nagington and Sandset 2020; Dodds 2021). I have shown that there is no strict boundary between moral considerations and evidence. The validity of either approach hinges as much on how much weight one places on the importance of biomedical or epidemiological factors as it does on moral, economic, and political values.

In this respect, we can see how certain ontological and normative commitments are present in the economic calculus underpinning the debates over PrEP’s value. The economic valuation method is premised on the problematisation of HIV transmission. If one considers the subject of transmission epidemiologically, PrEP is morally and economically inadvisable. However, a more dynamic route that omits the individual proves more productive. To make PrEP valuable to the state and individuals accustomed to calculating their risk, one must change how they perceive and act on risk.

Second, PrEP challenges the conventional distinctions between treatment and prevention upon which most HIV health services are based. This has been discussed elsewhere as a ‘purview paradox’, in which PrEP has deemed neither the responsibility of specialised HIV services, who tend not to see HIV-negative persons, nor the responsibility of general practitioners, who lack the expertise and resources to assess and monitor HIV treatments (Krakower et al. 2014; Hoffman et al. 2016). Particularly notable about PrEP in the English context was that these divisions were inscribed in policy, such that hybrid services were foreclosed. Between 2012 and 2022, the NHS was shaped by Cameron-era health reforms. Under Health Secretary Lansley, the UK health system was broken up into multiple semi-independent entities, each administering a different component of the healthcare system (Nicholas 2012; Greer 2016). In the area of HIV, prevention and treatment were delegated to two distinct services. The NHS had a centralised board responsible for HIV treatment as a specialised service, whilst HIV prevention was delegated to local health providers as part of routine sexual health services.

The NHS’s responsibilities in legislation and policy was to exclusively preoccupy itself with the treatment of persons with a verified HIV infection. This responsibility was demarcated from prevention and sexual health services delivered to HIV-negative persons. Though these categories employ biomedical language, HIV status is used to distinguish populations for HIV surveillance, remediation and, in the event of an infection, treatment. There is little consideration of the biological fuzziness of these categories at the biomolecular level, suggesting the style of problematisation is primarily epidemiological.

Policymakers attempted to respect this division of responsibility by splitting PrEP provision between the NHS and local authorities. What is important to note is that everyone agreed that PrEP was prevention and that the NHS could not administer PrEP under the 2012 regulations. For example, the proposed PrEP commissioning policy explicitly specified that the NHS was *not* the responsible commissioner for HIV preventive services*.* The PrEP service “(excluding the drug treatment) [would be commissioned] by local authorities, working in collaboration with NHS England…” who would be responsible for purchasing the drug (Foreman et al*.*, n.d, p. 13). Note in this case that policymakers were skirting a delicate line that allowed for a model of hybrid administration.

The proposed policy was sent to stakeholders who supported the proposed commissioning criteria but raised concerns about PrEP’s effects “on other HIV prevention strategies and commissioning arrangements” (NHS 2016a, p. 4). The NHS conducted a more comprehensive review of their responsibilities and concluded it could not commission PrEP because providing the drugs would also constitute a preventative service they were not empowered to provide without direction and additional resources from the Secretary of State (NHS 2016a).

### Making PrEP (almost) possible: National Aids Trust vs. NHS England

The National Aids Trust (NAT) sued the NHS, alleging discrimination and misrepresentation of the NHS’ legal obligations. Two judicial reviews ruled that the NHS was in fact responsible for PrEP. These reviews represent the molecularisation (Rose 2007) of legal judgement on public health matters. Whilst there were various legalistic arguments for why the NHS was responsible for PrEP, the argument that gained the most traction hinged on science. Specifically, the judges grappled with whether PrEP was HIV treatment or prevention. Their judgement that PrEP is treatment provided to otherwise healthy persons reflects a privileging of molecular arguments in policy.

Despite the rigidity of the Lansley reforms, the NHS admitted they were already involved in biomedical HIV prevention by providing routine HIV treatments. In a judicial review, they acknowledged offering post-exposure prophylaxis (PEP) and effective HIV treatment also prevents transmission. However, they argued these were still primarily treatments with prevention as a secondary benefit, maintaining the distinction between infected and uninfected. This biopolitical reasoning was necessary to justify their actions, which blurred the legislated distinction between treatment and prevention due to their biomedical effectiveness.

All parties agreed on the parallels between PEP and PrEP, leading to the NHS being held responsible for providing PrEP. The NHS could not convincingly deny its role in HIV prevention since it already provided treatments that could prevent HIV. Expert testimony from Sheila McCormack, the lead researcher on UK PrEP trials, confirmed the biochemical similarities between PrEP and PEP, arguing that neither medication prevents HIV transmission:

What they both do is prevent dissemination into an established infection. …the physiological benefits of the drugs are, in both cases, only present if transmission has already occurred. The only difference is that PrEP is taken before, as well as after, potential transmission occurs, whereas PEP is taken only after potential transmission has occurred. (*NAT vs. NHS England*, 2016 n.21).

The NHS concurred with this interpretation, noting that “at the molecular level [they are] the same whether the drug is provided as part of the PEP service or whether it is provided as part of the PrEP service” (*NAT vs. NHS England*, 2016 n.21). McCormack’s attestation to the biomedical efficacy of PrEP within the body seems to have convinced Justice Green that PrEP and PEP were indistinguishable.

What did not seem to convince Justice Green was the clinically meaningful distinction the NHS attempted to illustrate between pre-and post-exposure prophylaxis. For the NHS, the pre/post-distinction made all the difference because, with PEP, a clinician could assess the risk an infection occurred, more effectively than an antibody test. The clinician could not do so for PrEP because simply presenting as part of a *group* engaged in high-risk practices is insufficient (*NAT vs. NHS England*, 2016, n. 105). Hence, their argument hinged on what can be known about an early-stage infection. An epidemiological risk assessment is superior because there is no test sensitive enough to assess infection in the initial 72-h window where PEP is biologically active.

The court’s judgement, however, ruled that the clinical evaluation was, effectively, non-authoritative. As Justice Green writes,

the test of infection … is a test based upon pragmatic clinical judgment and not one of absolute scientific purity; (b) that in the case of those presenting following a high-risk event exposing them to HIV, clinicians are entitled to assume that they are infected (irrespective of the absolute scientific facts) (NAT vs. NHS England, 2016, n.107)

This passage illustrates the privileging of biomedicine over epidemiology in rendering a claim authoritative. Neither HIV tests and epidemiological surveillance make HIV infection directly visible. Both rely on abstractions to interpret infection. Epidemiological surveillance assesses HIV risk through sociological and statistical methods, whilst Fourth Generation HIV Immunoassays detect reactions of antibodies and antigens in blood samples. Neither method is necessarily better at forecasting HIV infection. Yet one is considered more seductive as a matter of *absolute scientific purity* and *fact,* whilst the other technique is equivalent to a guess*.*

Moreover, following the largely uncontested testimony of McCormack, Green could “see no material difference between PEP and PrEP that would justify a different treatment of PrEP relative to PEP”. Hence, he ruled that since the patient groups overlap, the treatment is materially the same, the practices are the same, the timing of treatment is immaterial, and both prevent infection; there was no reason to differentiate between PrEP and PEP. The conclusion, however, was not that PEP and PrEP were the same treatment, but that the subject: “PrEP is, by parity of reasoning to PEP, a treatment provided to those who should be assumed to be infected” (n.108). The appellate court supported Green’s characterisation, underscoring that PrEP formed a “seamless continuum” with PEP and HIV treatment and was, therefore, best administered by the NHS as a treatment to people who can be presumed to be as of yet uninfected but for whom an infection was incipient (*NAT R vs NHS England*, 2016).

Hence, the courts, in this instance, began molecularising HIV prevention. Firstly, the courts determined the distinctions between HIV-negative and positive were immaterial to the administration of HIV treatment. HIV treatment could now be administered to people who did not have a verified HIV infection if they believed they might become infected in the future. Moreover, they eliminated the role of the clinical appraisal, downgrading the clinical evaluation to an educated guess compared to the absolute scientific certainty required to assess if an infection had occurred. Hence, in this case, the argument is that biomedical knowledge is privileged. In its absence, clinicians ought to simply follow the guidance, which assumes members of former “risk groups” are now infected.

Contrary to others who have analysed the two judicial reviews on PrEP in England (Hurley 2019; Maine 2019), judges played a far more critical role in setting the stage for continuing the PrEP controversy and emergence of activism in the UK than previously suggested. The courts favoured a molecular approach to medical decision-making but did not mandate the NHS to adopt it. The appellate court ruled that whilst the NHS must provide PrEP as part of its services for adults with HIV, however the specifics of "reasonable provision" were left to the NHS’s discretion, reflecting a subtle shift in rationale rather than a change in the law.

## Representing PrEP

From 2017 to 2020, PrEP implementation stalled. Empowered to consider providing PrEP but not strictly required to commission it, the NHS proceeded with a more comprehensive review of its new responsibilities. PrEP was offered in a limited capacity via a clinical trial during this time. The PrEP Impact trial ran from October 2017 to June 2020 before PrEP became routinely commissioned in England. In the following section, I show how United4PrEP continued the debate sparked by the legal battle over this time. Whilst all advocate for universal PrEP provision, two networks emerge within the collaboration, each pursuing their implementation vision aligned with their thought styles. In the following sections, I discuss the differences in how these groups represent PrEP and its idealised subject. I illustrate a continuity of positions taken up in the judicial review, with one network taking on a reformist approach to PrEP integration, consistent with the NHS’ position above and the other taking a more radical approach reflective of the other position.

The way PrEP was represented was split along molecular and epidemiological lines. Whilst both styles of representation presented PrEP accurately, they represented it to conform with their preferred style. In the first instance, I will illustrate how the THT adopted an epidemiological style of representation focused on bodies and populations. This style reflects a more conventional somatic (Rose 2007) understanding of the body and self, in which HIV prevention is accomplished by actively keeping disease outside the body. It also represents the interests of authorities already entrenched in the extant HIV prevention dispositif.

### An epidemiological style of representation

The primary representative of the epidemiological style of thought is the THT. Founded in 1982, the THT is England’s leading advocate for PrEP provision. Based on a social worlds analysis of the materials the THT produced between 2016 and 2020, I found the THT tended to adopt positions that aligned closer to the NHS. As the UK’s first and largest HIV organisation, it has ingrained itself as a critical player in HIV-related governance, sharing language and conventions with healthcare institutions. Embracing a more conventional approach to HIV prevention, the THT views PrEP as a tool for HIV prevention, primarily for HIV-negative individuals at risk of transmission. They align themselves with clinical and governmental perspectives on HIV. Moreover, their understanding of PrEP’s function typically stops at its surface effects, reflecting an epidemiological thought style.

The THT is primarily an HIV advocacy organisation. Their mandate, to end HIV transmissions by 2030 and to provide HIV support and sexual health services until HIV is eradicated, permeates their advocacy work (THT 2023a). Hence, they aim to represent two patient communities: primarily people with HIV as well as those at risk of contracting HIV. This mandate positions the THT as representatives of the British people in general rather than any group. Moreover, how these people are represented by trustees and a board of directors suggests deep ties with British society. In addition to healthcare and HIV specialists, the THT boasts a list of around two dozen knighted, lorded and otherwise notable patrons, and their board of directors typically includes former members of parliament and representatives from industry (THT 2023b). In this respect, the THT can be described as representing the problematisation of HIV prevention through an insider lens that includes scientific, governmental, and economic specialists. The THT’s imbrication with British elites is reflected in the positions they took on PrEP provision. Though the THT became critical when the government and the NHS failed to live up to its own stated goal: to end HIV transmissions by 2030 (cf. Hancock 2019; THT 2020), the organisation is generally a supportive partner.

The alliances between the health system and the THT are not exclusive to their activism but frame their ontological positions on PrEP. Notably, the THT describe PrEP in a similar way as the NHS did above: as “a drug taken by HIV-negative people before and after sex that reduces the risk of getting HIV” (THT 2019). In this case, the temporality of prevention is important. HIV transmission, here framed as the act of sex undertaken by a demographic of HIV-negative persons at risk, is an event that can be predicted and managed. In this respect, one is meant to rationally appraise the utility of PrEP in relation to one’s own personal risk level and sexual acts. Further, they ascribe a less causal relationship to PrEP’s effects, arguing that PrEP is used to reduce risk (not eliminate it.)

Another way the THT echoes the NHS’s position is by stressing that PrEP does not form a seamless continuum with treatment but is exclusively preventative. The risk the individual is expected to visualise is *transmission.* Moreover, the transmission they are expected to visualise is as the movement of HIV between individual, self-contained bodies. This is reflected in how they describe how PrEP works by “block[ing] HIV if it gets into your body” (THT 2019). The risk here is somatic: Whilst the THT necessarily indexes HIV (a virus), the risk the individual is expected to visualise pertains to the risk posed by HIV to their whole body. In this respect, PrEP is described as analogous to a condom, which prevents HIV by acting as a barrier, preserving the integrity of two bodies by blocking the flow of infected fluids. The THT encourages people not to see certain things. Whilst PrEP prevents HIV, its functions *inside* the body are presented as unintelligible. In this respect, the THT is attempting to educate people to see PrEP through their lens, which includes teaching them not to notice or care about what PrEP is doing within the body.

### A molecular style of representation

The above representation differs from representations privileging molecular styles of thought. In contrast to the THT, the other main PrEP activist organisation, PrEPster, framed PrEP’s function and subject in relation to its biomedical effects. More closely aligned with biomedicine than the state or clinic, they represent PrEP as operating in the deep interiors of the body. In this style, treatment and prevention blur. Their subject and object belong to the ‘seamless continuum’ of biomedically enhanced bodies, irrespective of their HIV status or, as I will discuss below, choices or behaviour.

PrEPster agitated for PrEP implementation in England between 2015 and 2020. It was founded in London by Dr Will Nutland and Marc Thompson. Their identities as Gay men play an important in their activism, as does their HIV status (Mistlin 2021; Nutland 2015, 2020; Thompson 2024). Their professional experience is also noteworthy, as they are not only activists. Nutland holds a PhD in Public Health and has worked in the HIV sector since the late 80 s (Nutland 2015). Likewise, Thompson has held a number of roles in health organisations (Thompson 2024). It is through this combination of professional, sexual, and health identities that they felt compelled to start PrEPster. They created PrEPster in 2015 because they perceived the government and major HIV charities had “dropped the ball” on PrEP (Nutland 2017). Hence, Nutland and Thompson worked with other HIV prevention activists to fill this need.

PrEPster differs from the THT’s approach in four key aspects. First, it exclusively advocates for PrEP users. Second, it highlights PrEP’s alignment with HIV treatment, opposing the NHS’ position established in the judicial review. Third, it prioritises the direct impact of PrEP on HIV prevention over reducing risk. Finally, it encourages visualising PrEP’s effects on the body, embodying an alternative mode of thinking.

Whilst the THT and PrEPster both aim to represent those most at risk of contracting HIV and are in favour of PrEP, how these people are represented is starkly different. In addition to agitating primarily for PrEP, PrEPster (2022a) claims to be “led by…populations that are less likely to access PrEP education materials but are most in need of it”. Thus, PrEPster aims to represent and invite their subject to act as PrEP users. This community is also represented differently by activists. PrEPster’s most active members include both HIV-negative and positive individuals from affected communities, with significant experience in HIV charities or public health. Whilst PrEPster has a grassroots structure and a more adversarial relationship with the government compared to the THT, it remains well-integrated with health discourses, as its members are knowledgeable insiders in HIV and sexual health.

PrEPster’s composition and aims are reflected in how they represent PrEP. Firstly, in contrast to the THT, PrEPster emphasises the continuity between treatment and prevention. Echoing McCormack’s testimony above, PrEPster (2022b) defines PrEP as:

…a way of preventing HIV infection by taking a pill on an ongoing basis before sex and continued after sex. It’s taken by someone who doesn’t have HIV to prevent them from getting HIV. The PrEP pill is an antiretroviral drug – the same type of pill taken by someone who already has HIV to treat HIV.

PrEPster’s position on PrEP is nuanced. Whilst they also explain PrEP being taken before and after sex, they also stress its continuity with treatment as a drug taken on an ongoing basis. Likewise, they are more nuanced about the distinction between serostatus, stressing the shared identity of antiretroviral users.

Where the THT are more conventional in the approach to risk reduction premised on a division between HIV-negative and positive persons, PrEPster emphasises the *causal* relationship between HIV and PrEP’s mechanism of action. PrEPster emphasises the causal relationship by encouraging people to visualise how PrEP works inside the body.

[PrEP prevents] HIV from entering the cells and from replicating, which stops HIV from establishing itself and stops the person taking PrEP from becoming infected. For PrEP to work, there needs to be high enough levels of drugs in the blood to be protective against HIV (PrEPster 2022b).

PrEPster describes in detail what happens when people are exposed, inviting the reader to imagine what a sufficient concentration of the drug is doing *inside* their cells and blood. Moreover, PrEPster highlights that PrEP does not reduce the risk of interpersonal transmission but stops existing infections from taking hold.

PrEPster and THT are typically seen as allies in the PrEP controversy, collaborating from 2017 to 2020 to push the NHS for broader access to PrEP. However, their discursive positions reveal significant differences in how they conceptualise PrEP and the populations they represent. They have distinct goals and alliances, reflecting their role in perpetuating ongoing debate the courts did not resolve. Rose and Miller (1992) have discussed how advanced liberal governance is achieved at a distance from the state through their problematising activities. Indeed, as Hurley (2019) notes, whilst it was within their power to rule on the NHS’s responsibility, the courts could not close this debate as it would reflect judicial activism. So, the debate passed on from the courts to experts who could more effectively answer the question of their community’s needs. However, just like the courts privileged a particular ontology with respect to policy, PrEPster and the THT privileged two distinct ontologies, which shaped how PrEP was represented in evidence-based discourses, in part to fit with their goals and aspirations as activists imbricating of political and scientific representations, in the process.

## Representing the PrEP subject

The positions taken on PrEP shape the available subject positions. In the following section, I discuss how the two networks represent MSM as responsible PrEP users. A key aspect of the PrEP controversy that emerged following the PrEP U-turn concerned whether its primary intended users, gay and other MSM, truly needed PrEP. Mowlabocus (2019) notes a discursive shift following the U-turn. Before 2016, PrEP was framed as a public good comparable to vaccines, and like the birth control pill, it would permit MSM to exercise greater control over their sexual risk. After 2016, the morality of subsidising gay lifestyles, generally cast as reckless, would be brought into question.

The notion of the responsible biosexual citizen is further informed by the style of thought employed. The notion of the responsible subject, empowered to care for the self and others through acts of conscious choice (cf. Race, 2018; Rose 1999) and whose choices are shaped through a moral economy of hope (Rose and Novas 2005; Novas 2006) is more closely associated with an epidemiological style of thought. The molecularisation of sexual health, by contrast, offers a flattened conception of responsibility (Rose 2007), in which responsible sexual citizenship is shaped by a notion of bounded rationality, in which one’s right to be careless is underscored. In this respect, the differences are nuanced but radical, influencing how much attention a person is expected to accord to sexual health and when they need to make a conscious effort to prevent HIV.

### The epidemiological subject

The THT belong to a network of journalists and authorities predominantly from clinical medicine and government who integrate the experiential evidence of their patient population into a discourse that supports an epidemiological style of thought. This discourse is more recognisable than the conventional discourses of HIV prevention: MSM are compelled to act as prudential sexual entrepreneurs (cf. Adam 2005, 2006) who ought to prioritise the future health of the population over short-term, personal pleasures. These subjects are enjoined to adopt an epidemiological subject position, in which they are encouraged to act as active sexual citizens whose identity and responsibilities are filtered through epidemiological rationalities. Particularly, the ontological assumption that PrEP reduces the risk of transmission but does not prevent HIV on its own is reflected in how they use evidence and direct people to act in line with their vision of biosexual citizenship.

This network wants people to act conventionally: as prudential, enterprising subjects who conscientiously use all relevant and available HIV prevention technologies. This is best illustrated in how the THT responded to falling HIV rates. In 2017 and 2018, rates of HIV amongst MSM declined year-on-year for the first time since 1983, which many speculated was due to PrEP (cf.Boseley 2018; BBC 2017; Press Association 2018; Batchelor 2017; Matthews 2017). Consistent with their position above, representatives of the THT cited increased testing, early HIV treatment and the regular use of condoms and stressed, “PrEP-Optimism must not lead to complacency… we need to redouble our efforts, work harder and get to zero HIV transmissions” (Boseley 2018; BBC 2017). This position is further clarified by other clinicians who stressed the cause of HIV declines was likely “multi-factorial”, citing the importance of early testing and treatment for other STIs as equally responsible for declining HIV rates (Nwokolo et al. 2017). Note the subject is invited to see PrEP as a component of a toolkit that is equal to but not superior to other methods of HIV prevention. They are enjoined as active biological citizens, evoking hope for a future without HIV transmissions.

This network uses experiential evidence of MSM to normalise this subject position. For example, Harry Dodds shares his experience of taking PrEP:

Growing up gay in the shadow of HIV used to fill me with fear and anxiety. Now I’m on PrEP, I feel entirely confident that I’m protected from HIV and that the test is always going to come back negative. (Batchelor 2017)

This passage constructs Dodds as a representative of the empowered biosexual citizen. Identifying as gay, knowing his HIV status and using a combination of HIV preventative measures support this network’s position that those at risk cannot be complacent. Moreover, he explains how PrEP changed his feelings about HIV. “Removing that fear has been personally life-changing, and former anxiety has been replaced with hope for the future eradication of HIV” (Batchelor 2017). In this instance, experiential evidence is used to channel the conduct of its target community. Novas and Rose have illustrated how hope for a better biological future can be used strategically to engineer social and political change in the present (Novas 2006; Rose 2007; Rose and Novas 2005). In this instance, we can see how Dodds’ hope aligns him with a shared goal with this network: the future eradication of HIV through combination prevention.

This subject position is further normalised by pointing to vulnerable populations. A *Guardian* article introduces “Dan, a 25-year-old bisexual man … [who] was unable to find space on the NHS PrEP trial” (Newton 2018). He explains how this put him at a disadvantage because “buying PrEP privately can cost around £280 a year, which is unmanageable even while he is in full-time work”. Dan’s experience supports the THT’s concerns about reports of people being turned away from clinics, forcing them to buy PrEP privately (THT 2018). THT president Ian Green is quoted in the article, “We cannot wait until 2020 to do something about the current situation…We need a national programme as soon as possible to ensure PrEP is made available to everyone in England who needs it”. Dan clarifies how commissioning PrEP removes a crucial barrier that renders otherwise active biosexual citizens vulnerable. In this respect, Dan and Harry Dodds’ experiences reflect a fairly conventional way of normalising health behaviours.

Dan’s experiences complement Dodd’s active biosexual citizen position by illustrating how epidemiological barriers render him vulnerable. Dan explains,

…being turned away from the [Impact] trial was representative of a wider lack of information problem, and he considers himself lucky to even be aware of the drug….As an LGBT+, black, African individual, Dan falls into a number of particularly high-risk categories, and he believes the NHS should do a better job at reaching out to others like him. “At-risk people should be the priority,” he said. “I know people who are HIV positive who could have potentially not contracted the virus had PrEP been made available to them much earlier.” (Newton 2018)

Dan outlines the lack of information in his community, calls for greater outreach and gestures to the consequence of not being informed: contracting HIV. His experiences complement Dodd’s construction as empowered and confident in his contribution as a gay man to a future without HIV. In this respect, we can see how people are encouraged to reflect on themselves through community and biomedical notions of responsibility. Through its activities, this coalition attempts to reform individuals’ conduct to make room for PrEP without undermining the individual’s role as a conventionally prudent yet enterprising healthcare subject.

### The molecular subject

PrEPster and allied researchers enjoin people to think about themselves, their rights and responsibilities as biosexual citizens differently. They circulate experiential evidence about PrEP users to compel the British public to change how they think about HIV treatment and prevention entirely. What distinguishes this alliance from that above is that it directs people to think and act in line with a *molecular* style of thought. They attempt to install a novel set of norms consistent with knowledge derived from biomedicine rather than epidemiology. Notable differences in their style of representation are a focus on the present and personal desires, as opposed to the future. This focus on the present is complemented by a politics of causality, in which PrEP takes on a greater role than behaviour in HIV prevention. In this respect, the subject is compelled to take on distinct responsibilities inconsistent with the conventional figure of the enterprising yet prudent biological citizen.

Consider an editorial published by PrEPster founder Will Nutland (2020) in *The Guardian*. Nuland begins his editorial by reflecting on a train ride with his colleagues Phil and Marc in which he recalled a quote by gay rights activist Derek Jarman: “May you of a better future, love without a care”. He recalls how he thought of the line:

After meetings with sexual health activists from across Belgium, Marc took out the pill holder he keeps in his pocket when he travels and swallowed the medication he takes every day. Doing so reminded Phil and I that we should do the same, each of us gulping down a generic formulation of [PrEP]. This, as Jarman would say, is our better future.

This experience is used to invite people to think about themselves molecularly. Nutland establishes a continuity between Marc, who uses HIV medications to stay well and prevent transmissions, and Phil and Nutland, who use the drug to the same effect. The subject is defined not by their different HIV statuses but by their shared drug use.

They also interpret the epidemiological effects of PrEP differently. Nutland (2020) argues,

It is this combination of prevention methods – the testing and treatment of someone with HIV and the availability of PrEP to those most likely to be exposed to it – that led to a 71% fall in incidences of HIV among gay and bisexual men between 2014 and 2018, one of the most dramatic decreases ever registered.

The employment of this statistic is notable, as the THT interpreted the same rates radically differently. Whilst both groups employ statistical evidence, PrEPster infer a direct link between treatment and PrEP that “led” to these declines. Much like Justice Green’s judgement above, biomedical explanations are afforded more authority. Whilst a conventional reading of statistical data would emphasise a web of causal factors, as the THT outlines above, there is no web of causation for Nutland: Biomedical interventions alone *caused* measurable declines in HIV incidence. Moreover, they explicitly do not mention condom use or increased testing, emphasising the direct link between pharmaceutical prevention and HIV rate declines. The causal link and seamless continuum across treatment and prevention differentiates their molecular style of thought.

Nutland radically redefines HIV prevention by promoting a responsible *molecular* subject who prioritises effective treatment over HIV status, arguing that treatment better achieves prevention goals and aligns with current evidence. The above network used Dodd’s hope to align people with their aim of eliminating transmissions by 2030. Nutland, by contrast, argues his friends are already inhabiting a better future from the perspective of an imagined, past gay rights activist. “May you of a better future, love without a care…” (Jarman 2017) is utilised by Nutland as a call to change one’s thoughts and behaviours as a subject of HIV prevention. By taking PrEP, the reader is called to participate in an ethic where people do not need to preoccupy themselves with the imperative to prioritise HIV prevention over their sexual desires because PrEP takes care of it for them. Nutland’s position reflects a crucial difference in attention between the two styles of thought. Whilst the THT attempted to educate people not to care about what’s happening inside their bodies and to focus on their behaviour, PrEPster attempted to educate them not to care about their behaviour and to focus on “love”, reflecting friction between the two groups.

Jones et al. (2020) have already illustrated how PrEP activists use hope to draw a line from the “spectral histories of AIDS activism” through to PrEP as a way of directing conduct through the values of gay men. However, Nutland (2020) is not deploying hope for a future so people might act in the present (cf. Novas 2006). He offers a notion of hope, in which the future and past collapse into the present, reflecting a characteristic flattening of the ethical work one must undertake, which Rose (2007) associates with molecularisation. This way of compelling the subject to act in line with their problematisation is inconsistent with conventionally advanced liberal notions of calculative rationality but is consistent with notions of ‘bounded rationality’, increasingly popular in policy and PrEP discourses (cf. Pykett, Jones and Whitehead, 2017; Rendina 2015). Indeed, PrEP researchers argue people are not motivated not to use PrEP for HIV prevention but by immediate benefits, like pleasure, which are salient, effective and can be experienced in the present (Grant and Koester 2016). Nutland implicitly draws on this discourse when he argues people need not concern themselves with the future because we already inhabit it.

Nutland then calls on people to rely on different authorities. Nutland (2020) notes that gay men in London are privileged because they have insider knowledge. It is, “People who know how to navigate health services, are able to get to the front of a queue for a rationed service … or who have peers who tell them about PrEP, are the ones who benefit most from prevention technology”. But “it’s not just social advantages that influence PrEP take-up” Nutland (2020), writes,

…homophobia and racism in health services, stigma surrounding HIV, and a lack of awareness about available services may make it harder for these groups to access PrEP. Gender plays a role, too.

There are overlaps between Nutland’s barriers and those of the THT: homophobia, racism, stigma, and a lack of awareness amongst key populations are points of contact. However, he argues that people need access to *insider* knowledge to be better empowered.

The shift in authority and knowledge this network aims to encourage is reflected in how they use experiential evidence to normalise PrEP use. Another article discusses the experiences of Jack Ash, a PrEP user who changed his mind about PrEP when he moved to London. The article introduces him as a gay man who “came out in 1985, over a decade before effective treatment options”. Ash is a formerly domestic gay man who suddenly found himself single. He explains,

I had always used a condom. I’d lived through some of the worst years of HIV and AIDS, and I didn’t get it [PrEP], so I didn’t see why we needed it or, to be honest, why the NHS should fund it. (Siddons 2020)

The article goes on to paint a picture of a gay man whose concerns are out of place, out of date, and out of sync with the norms of the community. After his relationship ended, he moved to London, changed his mind about PrEP and started taking PrEP. PrEPster routinely constructs a figure of a gay man whose values are outdated (e.g. Pebody 2016) as a way of aligning subjects with their optimism for the present.

This article further paints the subject Ash is meant to represent as biased by moralising beliefs rather than scientific evidence. Quotes from Sheena McCormack and PrEPster bookend Ash’s story. McCormack says, “The science… is robust, but its effectiveness – over 99% according to some studies – did not convince NHS commissioners to make the drug widely available” (Siddons 2020). The article then links Ash’s attitudes to the concerns of this sceptical public,

Condom use is a common refrain among those sceptical of PrEP. The drug offers no defence against other sexually transmitted infections, and for those concerned that it encourages condomless sex— which some studies support – it’s a poor alternative …

Importantly, this example is used to dispel concerns about PrEP. The morality of this approach is underscored when Ash notes he took PrEP not to have more sex but because PrEP *guarantees* he will not get HIV. “I just sort of realised, I’ve got two kids, I’m here for the long haul, PrEP’s just a way of guaranteeing I won’t get HIV – it’s as much for their sake as mine” (Siddons 2020). In this respect, it is unclear if Ash is strictly represented or used as a cautionary tale; his experience transitioning from sceptic to devotee transforms, suggesting he is meant to compel people to change their minds concurrently as biological and sexual subjects.

Siddons (2020) also encourages a different way of thinking about one’s rights and responsibilities as a biosexual citizen. Rather than responsibilise gay people for using PrEP uniquely as HIV prevention, they advocate for the subject’s right to “slip up”. Phil Samba, a strategic lead for PrEPster, notes that,

Condomless sex among heterosexuals is treated as a fairly harmless slip-up, which we make provision for in the form of the morning-after pill. Sex between men doesn’t seem to engender the same duty of care (Samba, quoted in Siddons 2020)

Whilst earlier debates over PrEP used birth control parallels to highlight personal responsibility, framed in terms of rational choice (Mowlabocus 2019, pp.6–8). This is not Siddons and Samba’s argument. Whilst Ash reflects similar homonormative tropes present in earlier discourse, he is used to represent notions of a bounded subject, who sometimes slips up. By denaturalising condoms and drawing parallels with heterosexual practice, they install a different set of values and inequalities than the above network. Rather than point to informational or behavioural deficits, this network underscores the injustice of giving heterosexuals exclusive access to this biomedically enhanced right to slip up.

I have highlighted how this network diverges radically from the activities of the THT and their network. Whilst both groups advocate for removing barriers to PrEP’s full accessibility to everyone who needs it, they rely on different interpretations of the evidence and compel people to act in slight but meaningfully different ways. PrEPster’s deeper involvement with biomedicine allows them to make more radical claims about sexual liberation than the THT, whose ties to more established state healthcare aims appear to limit their claims. Most notably, this alliance attempts to engender a radically different subjectivity, in which people do not have to reflexively acknowledge their HIV status or assess their HIV risk in *the heat of the moment* to prevent HIV and stay healthy because that responsibility is transposed to PrEP, which they have taken in anticipation of said hot moment.

## Discussion

In 2020, PrEP was finally approved for routine commissioning as HIV prevention, available, free of charge through the NHS, to anyone who needs it, regardless of HIV risk. Whilst many on the outside were left wondering why implementation took so long to achieve so little (Kirby 2020), those with an insider account point to significant politicking, strategising and the deployment of uncertainties to delay and ration care (Dodds 2021; Nagington and Sandset 2020). This article offers a different approach to this debate, that highlights how contradictions in the administration of PrEP necessitates a series of reforms that resonated across the health system. In this respect, the decision not to provide PrEP was productive. Undoubtedly, the delay in PrEP implementation is unjust to everyone who could have benefitted from it in those six years. This delay is undeniably a clear and well-documented failure of the English health system. But what does this failure serve?

Contrary to humanistic interpretations of controversies, a governmentality approach considers failures as a transformative, if not productive force. Hence, critique does not end at pointing out that the UK government failed those who contracted HIV and neglected those at risk, nor at pointing out the benefits individuals or governments reaped whilst doing so. What this paper illustrates is the failures of the government to provide PrEP emerged from a challenge to the status quo of HIV services. Answering this challenge allowed for the installation of a novel way of doing HIV prevention. In the process, I noticed, at least in partiality, the emergence of a new way of thinking, being and acting relative to HIV. In place of the responsibilised subject enjoined to know themselves and exercise prudential choices towards a future free of HIV, a fallible subject, permitted not to think prudentially but to remain present in ‘the moment’ emerges.

Whilst United4PrEP collaborated and agitated for PrEP provision, in the name of marginalised populations, against a hostile Tory government, a less obvious conflict between integrationist and radical biosocialites was being fought. Two ways of conceiving the subject and object of HIV prevention, represented by two networks of experts and activists, emerged from their concurrent struggle for PrEP. I have illustrated how these networks dealt with the paradoxes identified with PrEP across HIV prevention services globally: a conflict between two styles of biopower vying for dominance over the subject, object and function and organisation of HIV prevention in the health system. In this respect, the UK PrEP controversy allows us to see what other health systems, by virtue of their successful integration of PrEP, cannot: the molecularisation of sexual health.

Whilst he does not address molecularisation directly, Kane Race (2018; 2016) argues that PrEP directly undermines the notion of the advanced liberal subject and consequently, is met with condemnation and moralism even from those who would benefit from it most. I have extended his ‘speculative foray’ into the problem of PrEP hesitancy by examining the conditions of its emergence in a particularly vivid case study and the effects of its failures.

There are three noteworthy transitions. Firstly, the PrEP controversy illustrates the hybridisation of political and scientific representation. Whilst patient organisations have become increasingly important in setting scientific agendas (Epstein 1996; Novas 2006; Rabeharisoa et al. 2014; Guta et al. 2014), this analysis illustrates how each style of thought empowered a different group of activists who in turn attempted to advocate for the same groups through their style of representation. Future researchers interested in healthcare activism should consider this dual meaning of representation. Relatedly, activists who occupied the centre of the debate were those who could identify concurrently as members of the relevant political and scientific communities. In this respect, we might consider how techniques of governing ‘through community’ and in the name of ‘life itself’ (Rose 1999, 2007) are bridged. Critical scholarship in HIV may benefit from attending to these hybridisations of identity-politics and science.

Second, this analysis underscores the relevance of ‘ontological multiplicity’ (Mol 2002) to governmental analyses. I have argued that PrEP’s biomedical promise has eclipsed the fact that, at a practical level, it did not make sense as HIV prevention. Specifically, this case study illustrated how different ways of seeing prevention failed to carry over from one area of the health system to the other. In light of this failure of ‘translation’ (cf. Rose 1999) from biomedicine to surveillance and service provision, the health system was changed to accommodate biomedical visions of HIV prevention. Whilst the effects of this shift are beyond the scope of this article, it is a promising area for future research.

The third and final emergence is the most interesting from a governmentality perspective: the molecular subject. The English debate points to the emergence of a novel biopolitical subject whose rationality and use of ‘biological capital’ (Foucault 2008) is radically distinct from the conventional neoliberal subject. This new way of thinking about MSM has the characteristic ‘flattening’ of subjectivity and, indeed, of the moral economies of healthcare associated with the molecularisation of the subject (Rose 2007). This flattened subject is also a bounded subject whose empowerment is *not* premised on thinking or acting rationally or deliberately in the moment, nor who is invested in long-term returns. Instead, sexual and social justice becomes contingent on the right not to think or judge. This shift in our conception of the subject represents a radical mutation in the subject of advanced liberal governmentality. Beyond England, features of this molecularisation of decision-making are increasingly present in healthcare (Djulbegovic 2021; Sandset et al., 2021; Will 2020) and in governmental programmes more generally (see Pykett, Jones and Whitehead, 2017).

Whilst the moralisation of preventative technologies is not novel, my analysis illustrates how overcoming the moralisation of PrEP results in the emergence of a new subject. Critical PrEP scholarship has argued PrEP allows a more nuanced form of pleasure-power to be exercised over its subjects (Dean 2015; Preciado, 2015; Sandset et al., 2021; Orne and Gall 2019). I have argued elsewhere that such interpretations of biomedical enhancement, those that uniquely focus on its disciplinary effects, do not do justice to the subject positions available and, thus, opportunities for resistance and experimentation (Lim et al. 2023). This analysis illustrates how subject positions are shaped by the possibilities offered by biomedical innovations and the constraints of historical precedent. PrEP emerges in a situation wherein the moral and economic rationale for PrEP is substantially weaker than its efficacy. For PrEP to be integrated, it had to be transformed into a good choice. To circumvent existing moral and economic arguments that ‘deviant queers’ were taking advantage of the health system (cf. Mowlabocus 2019) and to do so in a cost-efficient way, the subject was transformed into a person for which HIV infection was inevitable.

Consequently, we can see the emergence of two overlapping subject positions. Examining the distinct responsibilisation strategies employed by both networks helps us understand some of the contradicting requirements imposed on the subject of PrEP interventions. Examining PrEPster, in particular, illustrates how the figure of the responsible yet fallible subject is inscribed in HIV discourses and normalised through their activism. These claims run up against the responsibilisation strategies of the government and its allies, who attempted to integrate PrEP into existing programmes. Whilst the uptake of these discourses by individuals in England is beyond the scope of this paper, the extent to which these two groups collaborated without conceding a meaning to its subject may help us understand the contradictory requirements imposed on MSM that render PrEP a reluctant object (Race 2016; 2018).

## Data Availability

Not applicable.
